# Neuromodulation Strategies in Lifelong Bipolar Disorder: A Narrative Review

**DOI:** 10.3390/bs14121176

**Published:** 2024-12-08

**Authors:** Laura Bernabei, Beniamino Leone, Daniele Hirsch, Valentina Mentuccia, Alessia Panzera, Francesco Riggio, Loredana Sangiovanni, Valentina Piserchia, Giuseppe Nicolò, Enrico Pompili

**Affiliations:** 1 Department of Mental Health and Addiction, Psychiatric Service of Diagnosis and Care—ASL Rome 5, Colleferro, 00034 Rome, Italy; beniamino.leone@aslroma5.it (B.L.); daniele.hirsch@aslroma5.it (D.H.); vmentuccia5@gmail.com (V.M.); alessia.panzera@aslroma5.it (A.P.); loredana.sangiovanni@aslroma5.it (L.S.); giuseppe.nicolo@aslroma5.it (G.N.); 2 Department of Public Health and Infectious Diseases, Sapienza University of Rome, Piazza Aldo Moro, 100165 Rome, Italy; enrico.pompili@aslroma5.it; 3 Department of Mental Health and Addiction, Psychiatric Service of Diagnosis and Care—ASL Rome 5, Tivoli, 00019 Rome, Italy; francesco.riggio@aslroma5.it; 4 Department of Mental Health and Addiction, Centre of Mental Health—ASL Rome 5, Colleferro, 00034 Rome, Italy; valentinapiserchia@yahoo.it

**Keywords:** bipolar disorder, neuromodulation, TMS, tDCS, DBS, neurofeedback, ECT, VNS

## Abstract

Bipolar disorder is a debilitating psychiatric condition characterized by recurrent episodes of mania and depression, affecting millions worldwide. While pharmacotherapy remains the cornerstone of treatment, a significant proportion of patients exhibit inadequate response or intolerable side effects to conventional medications. In recent years, neuromodulation techniques have emerged as promising adjunctive or alternative treatments for bipolar disorder. We performed a narrative review, according to the Scale for the Assessment of Narrative Review Articles (SANRA) guidelines, to provide a comprehensive overview of the current literature on neuromodulation interventions in bipolar disorder across the course of lifespan. Specifically, it examines the efficacy, safety, and mechanisms of action of various neuromodulation strategies, including, among others, transcranial magnetic stimulation (TMS), electroconvulsive therapy (ECT), vagus nerve stimulation (VNS), deep brain stimulation (DBS), and it describes the therapeutic experiences across the different ages of illness. Additionally, this review discusses the clinical implications, challenges, and future directions of the integration, in clinical practice, of neuromodulation into the management of bipolar disorder. By synthesizing evidence from different studies, this review aims to inform clinicians, researchers, and stakeholders about the evolving landscape of neuromodulation treatments and their potential role in improving outcomes for individuals with bipolar disorder.

## 1. Introduction

Bipolar disorder (BD) is a complex, chronic and highly disabling illness characterized by severe and persistent mood fluctuations, during which depressive phases alternate with either manic phases (BD I) or hypomanic phases (BD II) [1]. In the text revision of DSM-5 (DSM-5TR), BD included BD I, BD II and Cyclothymic disorder [2].

Bipolar I disorder is characterized by various symptoms, including expansive mood, verbosity, excessive self-esteem, grandiosity, irritability, and significant reduction in the need for sleep, as well as prodigality and extreme disinhibition. Psychotic symptoms such as delusions and hallucinations occur in approximately 3/4 of manic episodes. Bipolar II disorder is primarily characterized by depressive episodes, interspersed with hypomanic episodes that never reach the severity of mania [1].

Beyond psychopathological symptoms such as anxiety and subthreshold symptoms, BD may be associated with a lack of psychosocial and cognitive functioning, manifested by attention, memory and executive deficits [3]. Moreover, global functioning may be compromised even during inter-episode periods, causing work, social, and relationship impairment [4].

It is estimated that it affects approximately 40 million individuals worldwide [5].

Following a trimodal distribution, different ages of onset have been suggested, although the average of BD onset is mainly around the age of 17.3 [6].

Generally, BD presents with a first depressive episode, both for BD I and BD II [7]. The depressive episode can have a variable duration but typically lasts much longer than the counter-polar episode. Since BD is also characterized by alternating periods of mood interspersed with inter-critical periods of euthymia, it is often misdiagnosed or underdiagnosed [1]

Over time, manic and depressive episodes, but especially depression, tend to become more frequent and this seems to be associated with greater impairment of global functioning [8].

Immune system dysfunction has a strong correlation with BD. Empirical research has confirmed that autoimmune diseases [9], chronic stress, cardiovascular disease [10], and metabolic disorders [11] are among the numerous inflammatory conditions associated with BD [12]. For example, studies on cytokines have shown that BD is linked to low-grade chronic inflammation, with pro-inflammatory cytokines being elevated during mood episodes [13].

There are multiple mechanisms that account for the reciprocal relationship between immune dysfunction and BD. Hypothalamic–pituitary–adrenal (HPA) axis over-activation, cytokine-induced changes in monoamines, elevated oxidative stress, pathological microglial over-activation, modifications to the microbiome–gut–brain axis, and immunological changes related to sleep are important mechanisms [13]. When it comes to treating BD, the inflammatory–mood pathway offers several innovative target options. Anti-inflammatory drugs have demonstrated a beneficial effect in a few proof-of-concept clinical trials for the treatment of BD [9].

One of the most complex problems related to the management of the BD is suicide. In fact, annually, patients with bipolar disorder present rates of attempted and completed suicide of the 3.9% and 1.4%, respectively; these rates are significantly higher than those of the general population, which are 0.5% and 0.02%, respectively. Bipolar patients experiencing a depressive episode or a mixed state have a higher risk of suicide than those experiencing just mania. Among bipolar patients, risk factors associated with depression for suicide attempts include multiple hospitalizations for depression and the presence of suicidal thoughts during depressive periods. Conversely, a prior suicide attempt has been shown to predict longer durations of depressive episodes, more severe depression, and a greater propensity for suicidal ideation [14].

Moreover, among BD patients, females exhibit a greater frequency of suicide attempts, whereas males demonstrate a higher incidence of completed suicides [15]. The same is true for young people [16].

Since BD is a complex disorder, international guidelines suggest an integrated management of the illness, in which pharmacological therapy is combined with psychotherapy and psychosocial interventions [17].

The pharmacotherapy for BD patients, from childhood to adulthood, is managed according to the phase of the illness; in fact, major international guidelines recommend a specific therapy for the manic/mixed state, for the depressive state, or for the maintenance of euthymic states (Table 1) [5,18,19]; Otherwise, the goal of pharmacological treatment is to reduce the intensity of the current episode and control the number and the severity of future episodes. In this way, typical antipsychotics and benzodiazepines (BDZs) are not recommended because they may be prescribed only for the acute management of agitation but not for long-term use [5].

Within the psychotherapy for BD, Cognitive and Behavioral Therapy and Psychoeducational treatments are the most cited and effective interventions [20].

Although approved treatment protocols for BD (both psychopharmacological and psychosocial), already exist and are routinely used, on the one hand, pharmacological approaches for BD may not consistently yield the desired results and may carry safety concerns, but on the other hand, nonpharmacological interventions (such as CBT, psychiatric rehabilitation, psychoeducation, etc.) may not always be effective, easily accessible, or scalable [21] (Table 2). Therefore, there is an urgent need for the development of new therapeutic interventions that can be tailored to the specific needs of the patient. For example, neuromodulation, in its various forms, can be considered an important tool to be used within the integrated treatment of BD [22].

## 2. Aim

Neuromodulation treatments have emerged as promising interventions for different neurological and psychiatric disorders [23,24,25,26]. As healthcare providers seek to optimize patient care, the process of referring individuals to neuromodulation therapies demands careful consideration and adherence to best practices. This paper aims to (i) delineate the major neuromodulation approaches offering updated information on innovative adjunctive treatments for BD that are implemented in clinical practice; (ii) define the essential steps involved in effectively sending patients for neuromodulation treatments; and (iii) ensure comprehensive evaluation, appropriate selection, and seamless transition into the therapeutic regimen, aiming to support clinicians in their efforts to enhance functional recovery for patients. Although a substantial number of studies in the literature focus on the application of neuromodulation techniques in BD, there is currently no article that discusses both invasive and non-invasive neuromodulation techniques while describing their application in clinical practice.

## 3. Methods

This narrative review aims to describe the current neuromodulation strategies used in the clinical practice of bipolar disorder treatment. To conduct this narrative review, the authors followed the SANRA (Scale for the quality Assessment of Narrative Review Articles) guidelines [27] and supplemented them with the SWiM checklist [28] to enhance the information related to the synthesis process and improve the overall quality of the information provided. This research was conducted using the PICO framework to ensure a structured and focused approach: (i) population (P): patients diagnosed with bipolar disorder (BD) according to DSM-5TR criteria were included; (ii) intervention (I): the primary interventions examined were neuromodulation strategies, invasive and non-invasive; then, the different approaches were detailed: TMS, ECT, VNS, tDCS, BS, NF, TES, TUS; (iii) comparison (C): where applicable, comparisons were made with sham interventions, other treatments, or standard care; (iv) outcome (O): the main outcomes assessed included the improvement in clinical symptoms, the remission rates, cognition, global functioning, or the quality of life measures. Additional methodological details are provided to clarify the search strategy, inclusion/exclusion criteria, and data synthesis process.

An extensive research study was conducted to identify suitable papers (LB, VP, VM, BL, DH, EP). The references cited in this review were sourced from thorough searches of electronic databases including Embase, PubMed/MEDLINE, EBSCO, and APA PsycINFO. The following keywords were used in combination with “Bipolar Disorder”: transcranial magnetic stimulation (TMS), electroconvulsive therapy (ECT), vagus nerve stimulation (VNS), deep brain stimulation (DBS), neurofeedback (NF), transcranial Electrical Stimulation (TES), transcranial Direct Current Stimulation (tDCS), and Transcranial Ultrasound Stimulation (TUS). Only studies written in English and published in peer-reviewed journals from 1985 (the year TMS was first proposed by Anthony T. Barker as a non-invasive method to study the functional characteristics of the motor corticospinal pathways in humans) [29] to April 2024 for TMS, from 1946 to April 2024 for ECT, from 2000 to April 2024 for VNS, DBS, NF, TES, and TUS, and from 1996 to April 2024 for “non-invasive neuromodulation” and “invasive neuromodulation” were included. Priority was given to published reviews, and meta-analyses to offer a comprehensive and current summary of the clinical applications of neuromodulation strategies in patients with BD. When necessary, research articles, case reports, editorials, mini-reviews, practice guidelines, short communications, and open-label studies were cited. An additional in-depth search was conducted based on the reference lists of the articles of interest. All information was narratively summarized, and the main results were discussed within the dedicated paragraphs. From a total of 25,540 studies, 14,945 duplicates were removed. An additional 10,226 papers were excluded prior to screening as they did not meet the eligibility criteria (e.g., studies conducted on animals, rats, mouse, healthy controls, etc.). The remaining 369 studies were further screened, and 249 papers were excluded as they did not meet the PICO criteria (the exclusion criteria included Parkinson disease, Major Depression, etc.). A total of 120 papers were reviewed, and 25 were selected for inclusion in this narrative review.

## 4. Neuromodulation Treatments

Neuromodulation techniques encompass a range of interventions, which have seen rapid development in recent years, aimed at modulating neural activity to treat various neurological and psychiatric disorders, including BD [24,25].

These techniques use different devices, and they can generally be broadly categorized into *invasive* and *non-invasive approaches*.

Invasive neuromodulation techniques typically involve the implantation of electrodes or devices directly into specific brain regions or nerves. Examples include deep brain stimulation (DBS) and vagus nerve stimulation (VNS). Non-invasive techniques, on the other hand, do not require surgical intervention and can be applied externally to the body. Examples include photobiomodulation (PBM), neurofeedback (NF), electroconvulsive therapy (ECT), and transcranial stimulation with its different protocols [transcranial Magnetic Stimulation (TMS), tetha-burst stimulation (TBS), transcranial Electrical Stimulation (TES), transcranial Direct Current Stimulation (tDCS), and transcranial Ultrasound Stimulation (TUS).

The primary goal of neuromodulation in BD is to regulate neural circuits implicated in emotional regulation, thereby alleviating symptoms and enhancing mood stability [30]. Moreover, a mounting body of research has affirmed that these neuromodulation techniques can modulate chronic inflammation affecting BD patients while concurrently alleviating psychiatric symptoms [30,31,32]. Neuroplasticity, which encompasses dynamic alterations in neuronal connections in the central nervous system, serves as the foundation for various brain functions, including cognitive processes such as learning and memory. In recent years, it has become increasingly evident that neuroplasticity dysfunctions are involved in several neuropsychiatric disorders, and many studies show that neuroplasticity can be induced in humans using non-invasive brain stimulation techniques [33].

The mechanisms of action underlying neuromodulation techniques vary depending on the specific method employed. For example, DBS involves the delivery of electrical impulses to targeted brain regions, modulating neuronal activity and neurotransmitter release. Similarly, VNS modulates neural activity via the stimulation of the vagus nerve, which sends projections to different brain regions involved in mood regulation.

However, neuromodulation techniques also offer distinct advantages. They can provide targeted and personalized treatment approaches, particularly for patients who have not responded to traditional pharmacological or psychotherapeutic interventions. Furthermore, these techniques are generally well tolerated, with very few serious adverse effects reported in clinical trials [26].

**Figure 1:**
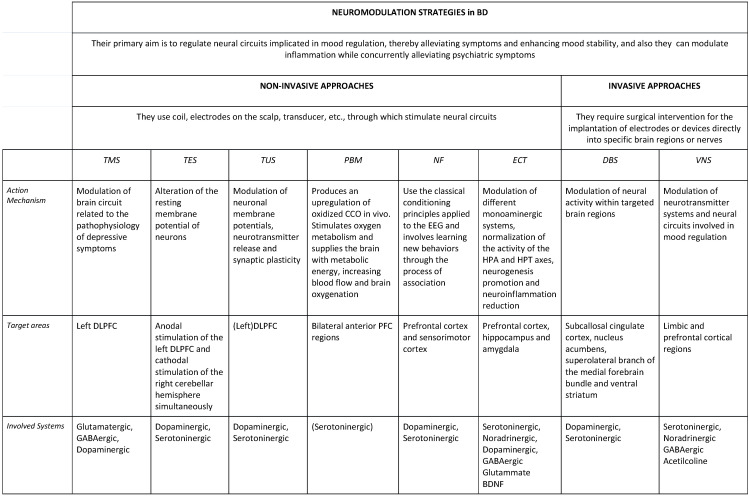
Differences among the neuromodulation approaches. Note: TMS (Transcranial Magnetic Stimulation); TES (Transcranial Electrical Stimulation); TUS (Transcranial Ultrasound Stimulation); tDCS (Transcranial Direct Current Stimulation); PBM (Photobiomodulation); NF (Neurofeedback); ECT (Electroconvulsive Therapy); DBS (Deep Brain Stimulation); VNS (Vagus Nerve Stimulation); DLPFC (Dorso Lateral Prefrontal Cortex); CCO (Cytochrome C Oxidase); EEG (Electroencephalogram); HPA (Hypothalamus–Pituitary–Adrenal); HPT (Hypothalamus–Pituitary–Thyroid). Level of evidence qualifying studies:—I high-quality, multicenter or single-center, randomized controlled trial with adequate power; or systematic review of these studies;—II lesser-quality, randomized controlled trial; prospective cohort or comparative study; or systematic review of these studies;—III retrospective cohort or comparative study; case–control study; or systematic review of these studies;—IV case series with pre/post-test or only post-test;—V expert opinion developed via consensus process; case report or clinical example; or evidence based on physiology, bench research, or “first principles”.

Evidence regarding the efficacy of neuromodulation techniques in BD is still evolving. While some studies have reported significant reductions in depressive and manic symptoms following neuromodulation therapy, others have yielded more mixed results [34]. Further research, including larger randomized controlled trials with longer follow-up periods, is needed to establish the efficacy of neuromodulation as a treatment for BD conclusively (Figure 1).

## 5. Non-Invasive Approaches

### 5.1. Transcranial Magnetic Stimulation (TMS)

The past twenty years have seen significant progress in the development of brain stimulation methods.

In a letter to Editor, Barker et al. (1985) introduced TMS as a tool to investigate the functional characteristics of the motor corticospinal pathways in humans [29], and its use spread quickly. Although it is used as an investigative tool, TMS has shown great potential when used to modulate neural circuit activity [30].

In fact, TMS modulates neurotransmitter systems by increasing cortical excitability and synaptic plasticity. It particularly influences the glutamatergic and GABAergic systems, enhancing excitatory and inhibitory balance. Additionally, TMS has been showed to affect the dopaminergic system, potentially increasing dopamine release in targeted areas.

TMS can be appreciated for its versatility. The different protocols can vary based on (i) the location of stimulation (i.e., the side of the stimulation: right-sided vs. left-sided, bilateral vs. unilateral, etc.), (ii) duration, (iii) stimulus frequency and (iv) intensity.

Among these advancements, the establishment of repetitive transcranial magnetic stimulation (rTMS) targeting the left dorsolateral prefrontal cortex (DLPFC) stands out as the most notable, offering a safe and effective treatment for depression with minimal side effects [35].

A standard protocol requires the administration of high-frequency stimulation (10 Hz) for 37.5 min for 5 days per week for at least 6 weeks. However, the accelerated protocols suggest that repetitive TMS (rTMS) may also be effective using a shorter time (e.g., 18.75 min protocol) or a low frequency (1–2 Hz) for a 4-week treatment [36,37,38]; rTMS, which encompasses high-frequency rTMS (HF-rTMS), inducing excitatory effects at ≥5 Hz, low-frequency rTMS (LF-rTMS), inducing inhibitory effects ≤ 1 Hz, theta-burst stimulation (TBS) and accelerated intermittent theta-burst stimulation (aiTBS), has been mainly employed in the treatment of Major Depressive Disorder (MDD) [39]. The stimulation of DLPFC impacts connected neural circuits, including the fronto-striatal and fronto-limbic pathways, which are involved in mood regulation and executive function. rTMS is the most widely used protocol for the treatment of unipolar depression and, recently, for the treatment of BD too [39,40,41]. Its therapeutic mechanism of action is mainly focused on the remodulation of brain circuits involved in the maintenance of depressive symptoms. In fact, most research with rTMS focuses on depressive symptom of BD, while little is known about its effects in mixed or maniac states [38].

The other specific rTMS protocol, TBS, employs short bursts (three pulses at 50 Hz) delivered at 5 Hz. Lastly, deep TMS (dTMS) is a particular TMS that uses the H coil held inside a padded helmet to achieve deeper and large brain volumes [30].

The predictors of response to TMS treatment are different. Some studies suggest that the efficacy of treatment response could be influenced by the type of depressive symptoms experienced by the patient (*cognitive–affective* versus *somatic symptoms* or *emotional* versus *bodily*), some antidepressant or antihistamines taken during TMS or the illness duration (months or years of illness) [41].

Numerous studies confirm the clinical efficacy on depressive symptoms of rTMS in adult BD [40] moreover, it has shown to be safe, well tolerated (while some studies reported greater energy, insomnia, irritability, anxiety and suicidal attempt), and characterized by a low rate of drop-out [38,42,43]. However, clinical studies are characterized by a small sample size, although randomized control trials are high compared to open-label studies.

In the pediatric population, the existing research is very limited, but in recent years, the promising results obtained in the treatment of adults BD have led researchers to advocate for the use of TMS even among younger patients, especially in cases where traditional treatments are inaccessible, poorly tolerated or ineffective [44].

### 5.2. Transcranial Electrical Stimulation (TES)

Transcranial Electrical Stimulation (TES) includes a range of neuromodulation techniques that involve the application of electrical currents to the scalp with the aim of modulating neural activity in the brain [45]. These techniques include transcranial Direct Current Stimulation (tDCS), which applies a direct electrical current of low intensity with an anode and a cathode; transcranial pulsed current stimulation (tPCS); transcranial alternating current stimulation (tACS), which applies an oscillating, sinusoidal electrical current of low intensity on a specific brain area; and transcranial random noise stimulation (tRNS). Further sub-categories reflect differences in the aspects of electrode montage, waveform and outcomes [46,47].

TES has gained significant attention in neuroscience research and clinical applications due to its potential to enhance cognitive function, facilitate neuroplasticity, elicit long-lasting alterations in cortical excitability, and treat neuropsychiatric disorders, including BD [46].

The primary purpose of TES in BD is to modulate neural circuits implicated in mood regulation, with the goal of alleviating depressive and manic symptoms and promoting mood stability.

Additionally, TES may induce neuroplastic changes in brain regions associated with mood regulation, such as the prefrontal cortex and limbic system [47,48,49]. Cognitive functioning is involved in many psychiatric disorders; the observed impairment can be profound or relatively mild [50], and its amelioration through TES could lead to improvement in daily life functioning.

The mechanisms underlying the therapeutic effects of TES are not fully understood but are thought to involve changes in neuronal excitability by altering the resting membrane potential of neurons [51], and synaptic plasticity. In fact, TES aim to directly modify brain function by passing low- or high-amplitude electrical currents using an electrode placed on the scalp [45].

Within the TES techniques, Transcranial Direct Current Stimulation (tDCS) involves applying a low-intensity electrical current on the scalp via electrodes, with the aim of modulating neuronal activity in the targeted brain regions. tDCS shifts resting neuronal membrane potential toward anodal or cathodal polarization without ever exceeding the depolarization threshold of neurons due to the weak and constant polarization [52]. Anodal stimulation, characterized by the application of a positive current, is thought to depolarize neurons, thereby increasing their firing rates, whereas cathodal stimulation, involving a negative current, is believed to hyperpolarize neurons, leading to decreased excitability. This current flow between the electrodes stimulates neurons, producing physiological and behavioral modification.

Such protocols simultaneously involve anodal stimulation of the left DLPFC and cathodal stimulation of the right cerebellar hemisphere to improve neuropsychological and neurological functioning [49,50].

These effects can persist beyond the duration of stimulation and may induce long-term changes in synaptic plasticity [53]. For example, tDCS is believed to modulate the resting membrane potential of neurons, thereby altering their firing rates and influencing neurotransmitter release. Begemann et al. (2020) [54] in their meta-analysis, found a positive and significant effect of tDCS on working memory and in attention/vigilance in patients affected by neuropsychiatric and psychiatric disorders. Numerous studies highlighted a significant improvement on cognition in BD patients and sleep quality in euthymic BD patients, too. In particular, prefronto-cerebellar tDCS contributes to improving visuospatial memory, executive functions and neurological soft signs in euthymic adult BD [49,55]. Sleep quality is increased after a prefronto-cerebellar tDCS delivered for 20 min per day over three consecutive weeks in a euthymic adult BD [48].

However, the efficacy of tDCS in BD is still under investigation. Many studies suggest that tDCS has effects on BD depressive phases, although data on tDCS in manic or hypomanic phases are scarce.

A case report found that tDCS as an add-on pharmacotherapy may improve manic symptoms in patients with BD. More research is needed to assess the potential efficacy of tDCS in patients with hypomanic and/or manic symptoms [26].

In the young BD population, tDCS showed promising results, but definitive outcomes are still unknown [56,57].

Moreover, given the different protocols used in the literature, it is not possible to draw clear conclusions.

In terms of regulatory approval, the U.S. Food and Drug Administration (FDA) has granted clearance for certain tDCS devices and specific indications. However, it is essential to note that the FDA’s regulatory oversight primarily pertains to the safety and efficacy of medical devices rather than specific treatment protocols. As such, there is ongoing research to establish standardized protocols and optimize the efficacy of tDCS for various clinical applications [58].

Despite its potential benefits, TES has several limitations. One limitation is the variability in treatment response observed among individuals, with some patients experiencing significant symptom improvement while others derive minimal benefit. Additionally, the optimal parameters (e.g., stimulation intensity, duration, and electrode placement) for TES in BD have yet to be established, leading to challenges in standardizing treatment protocols. Moreover, the long-term effects of TES on mood stability and relapse prevention in BD remain unclear [47].

However, TES techniques also offer several advantages. They are non-invasive and relatively well tolerated, with few serious adverse effects reported in clinical trials. Furthermore, they can be administered as an adjunctive therapy to pharmacological and psychotherapeutic interventions, potentially enhancing treatment outcomes for individuals with BD [50,59]

The efficacy of TES in BD is still being investigated, with mixed findings reported in clinical trials [60]. While some studies have reported significant reductions in depressive and manic symptoms following TES therapy, others have yielded more modest or inconsistent results.

Further research, including large-scale randomized controlled trials with longer follow-up periods, is needed to elucidate the therapeutic efficacy and optimal parameters of TES in BD conclusively [33].

### 5.3. Transcranial Ultrasound Stimulation (TUS)

Transcranial Ultrasound Stimulation (TUS) is a non-invasive neuromodulation technique that involves the application of low-intensity ultrasound waves to specific regions of the brain, modulating the activity of the primary somatosensory cortex [61]. Its use is relatively recent.

For its properties, TUS was first used with neurological patients. The first research study, in 2013, showed improvement in scores of mood and pain evaluation in chronic pain patients [62].

The mechanism of action of TUS in BD is not fully understood, but it is hypothesized to involve several processes, owing to its excitatory or inhibitory activity, depending on the sonication parameters applied [63].

Ultrasound waves penetrate the skull and reach the brain with a frequency >20 kHz, where, interacting with membranes and mechanosensitive channels, they may influence neuronal activity by modulating neuronal membrane potentials, neurotransmitter release, and synaptic plasticity, thereby inducing a neuronal signaling cascade [64]. In mouse models studies of depression, it has emerged that Low-intensity Transcranial Ultrasound Stimulation (LITUS) of the prefrontal cortex (PFC) or ventromedial prefrontal cortex (vmPFC) weakened depressive-like behaviors. Further evidence for a common BDNF signaling pathway triggered by ultrasound stimulation comes from the observation that LITUS increased BDNF levels in the hippocampal regions of normal mice. Moreover, LITUS increased adult hippocampus neural stem cell proliferation and neurogenesis; the latter is an important mechanism underlying depression and is a side effect of antidepressant medications [63]. Therefore, TUS may stimulate neurogenesis and enhance neuroplasticity, processes that are disrupted and/or altered in mood disorders such as BD.

Unlike other transcranial stimulation techniques, TUS can stimulate both cerebrocortical surface and deep brain structures [64].

Despite its potential, TUS has certain limitations. One limitation is the lack of a precise targeting of brain regions, as the therapeutic effects of TUS depend on the accurate delivery of ultrasound waves to specific neural circuits. Additionally, the optimal parameters for TUS therapy in BD, including waveform, intensity, and duration of stimulation, have yet to be fully elucidated. Furthermore, the long-term safety profile of TUS for BD treatment remains to be established.

TUS has garnered interest as a potential therapeutic intervention for various neurological and psychiatric disorders, including BD [65]. The primary objective of TUS in the context of BD is to modulate neural activity in brain regions implicated in mood regulation, with the aim of ameliorating cognitive symptoms and enhancing mood stability [62,64,66].

However, TUS also offers several advantages. It is a non-invasive technique that does not require surgical implantation or anesthesia, making it well tolerated and suitable for repeated sessions. Moreover, TUS has the potential to modulate neural activity with high spatial resolution, allowing for precise targeting of brain regions implicated in BD pathophysiology [67].

The efficacy of TUS in BD is still under investigation. While preclinical studies and small-scale clinical trials have shown promising results in terms of reducing depressive and manic symptoms, larger randomized controlled trials with long-term follow-up are needed to conclusively confirm its efficacy and safety as a treatment for BD.

### 5.4. Photobiomodulation (PBM)

Photobiomodulation (PBM), also known as low-level laser therapy (LLLT) or cold laser therapy, is a non-invasive therapeutic technique that utilizes low-level light sources (usually in the red or near-infrared spectrum) to modulate cellular functions. It has several effects, such as boosting ATP synthesis, repairing damaged hypoxic cells, altering target tissue function, and stimulating mitochondrial metabolism [67,68]. It is precisely because of these properties that PBM has attracted attention in various medical fields, including neurology and psychiatry. In particular, Transcranial Infrared Laser Stimulation (TILS), based on photobiomodulation, is a new non-invasive form of low-level light therapy which uses an infrared laser within the range of 808 nm and 1064 nm and has been applied for its potential therapeutic effects in mood disorders such as BD [69].

The mechanisms of action of PBM involve the absorption of photons by mitochondrial chromophores, leading to increased adenosine triphosphate (ATP) production, modulation of reactive oxygen species (ROS) levels, and upregulation of various cellular signaling pathways. Some studies highlighted the presence of various mitochondrial alterations in psychiatric disease, included BD [69,70].

Additionally, PBM has been shown to promote neurogenesis, enhance synaptic plasticity, and reduce inflammation [71], all of which may contribute to its therapeutic effects in BD [72].

The primary purpose of PBM in the context of BD is to alleviate depressive and manic symptoms, improve mood regulation, and potentially stabilize mood fluctuations. This is achieved through the modulation of cellular processes, particularly in the brain, which are thought to play a role in the pathophysiology of BD [68].

In the research study by O’Donnel et al. [69], the first that examines how TILS improves cognition in BD patients, BD subjects (*n* = 5) received 1 min invisible laser light at 1064 nm and a dim red guiding light of 560 nm alternating every minute between the right and left anterior PFC for 10 min per session. At the end of treatment, they showed an enhancement in cognitive frontal functioning.

Despite its promising potential, PBM also has limitations and challenges. One limitation is the lack of standardized protocols regarding treatment parameters such as wavelength, intensity, and duration of exposure. Additionally, the evidence supporting the efficacy of PBM in BD is still preliminary, with relatively few well-designed clinical studies available [73].

However, PBM offers several advantages as a therapeutic approach for BD. It is non-invasive, well-tolerated, and associated with minimal side effects, making it suitable for long-term use.

Furthermore, PBM can be easily administered and integrated into existing treatment regimens, including pharmacotherapy and psychotherapy [74].

Regarding its efficacy in BD, while preliminary studies have shown promising results in terms of mood improvement and symptom reduction, more rigorous research is needed to establish its effectiveness and determine optimal treatment protocols. Future randomized controlled trials with larger sample sizes and longer follow-up periods are warranted to further elucidate the role of PBM in the management of BD.

### 5.5. Neurofeedback (NF)

Neurofeedback (NF) is a form of biofeedback that aims to modulate brain activity by providing real-time feedback on brainwave patterns, typically through electroencephalography (EEG). This technique has gained attention as a potential intervention for various neurological [75] and psychiatric conditions, including BD.

NF involves training individuals to self-regulate their brain activity by presenting them with real-time information about their neural functioning, often in the form of auditory or visual signals. Through repeated sessions, individuals learn to modulate their brainwave patterns, aiming to achieve desired states associated with improved cognitive and emotional functioning [76]. Neurofeedback operates by detecting and recording the patient’s brain signals using methods like EEG, fMRI, or near-infrared spectroscopy and then immediately feeding relevant information back to the participant. When the signal meets a specified target, the participant receives a reward, demonstrating that autonomic functions can be modified through operant conditioning. Building on this concept, operant conditioning has been used to enhance sensorimotor EEG rhythms, effectively reducing seizure frequency in epileptic patients. Positive outcomes have also been observed in treating attention-deficit hyperactivity disorder by training to increase alpha and decrease theta activity [77]. There are several objectives that can be achieved with this treatment: (i) *symptoms*: NF is utilized with the aim of reducing symptoms associated with BD, including mood swings, cognitive impairments, and emotional dysregulation; (ii) *enhanced self-regulation*: the training aims to improve self-regulation of neural activity, potentially leading to better mood stability and overall functioning, including the reduction in impulsivity behaviors [78]); (iii) sleep improvement: the training may modulate the amount of sleep (too much or not enough) in different phases of BD [9,75].

NF uses different protocols for specific therapeutic needs. For example, the therapist may apply from two up to twenty-one electrodes in different brain areas in reference to 10–20 system; other differences include (i) the frequency band trained [e.g., delta (1–4 Hz); theta (4–8 HZ); alpha (8–12 Hz); sensorimotor rhythm SMR (12–15 Hz); low-beta (13–21 Hz); high-beta (21–35 Hz); gamma (35–45 Hz); theta/beta ratio; etc.)]; (ii) the different location of electrodes (Fz, Cz, etc.); (iii) the different conditions of the subject (e.g., closed or open eyes, simultaneously use of a virtual reality headset); (iv) the different tasks to be performed (relaxation, problem solving, puzzle, etc.).

NF operates on the principle of neuroplasticity, the brain’s ability to reorganize and adapt in response to experiences. By providing feedback on brain activity, individuals can learn to modulate their neural patterns, leading to changes in brain functioning over time [79].

There are a few limitations regarding its use. The effects of NF may not generalize well beyond the training setting, limiting its long-term efficacy (generalizability). NF training requires multiple sessions over an extended period, making it resource-intensive and potentially inaccessible to some individuals.

On the other hand, NF has great advantages. Firstly, NF is non-invasive and does not involve the use of medication, making it a potentially attractive option for individuals seeking non-pharmacological interventions. Secondly, training protocols can be tailored to target specific symptoms or neural networks implicated in BD, allowing for individualized treatment approaches. The typical neurofeedback (NF) task is explicit, meaning participants are aware that they are receiving neural feedback and are given clear instructions on the task. It is also usually continuous, with feedback being calculated for each collected brain signal and presented to the participant in real-time [80] (However, NF protocols can differ in these key aspects, which allows them to be tailored to target specific processes. When the clinicians use an implicit feedback protocol, participants receive rewarding feedback without knowing its source or being instructed to regulate it. They may simply watch target-related cues or perform an unrelated task, such as pressing a button. This approach minimizes confounding factors like effort or cognitive load, which is especially useful when isolating neuromodulation effects on attention and executive functions. Implicit neurofeedback may also be more suitable for individuals with limited cognitive resources, such as those with severe attention deficits, dissociative symptoms, or very young or elderly populations. Additionally, neurofeedback offers the advantage of reinforcing specific mental imagery in patients [81].

Research on the efficacy of neurofeedback in BD is still in its early stages. While some studies have reported promising results in terms of symptom reduction and mood stabilization, larger-scale randomized controlled trials are needed to establish its effectiveness as a standalone or adjunctive treatment for BD [80].

### 5.6. Electroconvulsive Therapy (ECT)

Electroconvulsive Therapy (ECT) is a complex treatment administered by a specialized team within a dedicated hospital setting. It offers potent efficacy and a rapid onset of action, even after alternative treatments fail. ECT has an exceptionally broad therapeutic spectrum, with evidence of benefits in unipolar and bipolar disorder (including the acute treatment of depressive and manic episodes, psychotic subtype, as well as relapse prevention), catatonia, schizophrenia, schizoaffective disorder, Parkinson’s disease, epilepsy, status epilepticus, repetitive self-injury in autism, tardive dyskinesia, and neuroleptic malignant syndrome [82]. ECT was introduced in 1938 in Italy, and its technology and treatment delivery have since seen major improvements. Despite nearly nine decades of use in psychiatry demonstrating its efficacy, the precise mechanisms by which ECT exerts its therapeutic effects and its cognitive side effects are not fully uncovered and are still being studied, in particular, the effects of electrical stimulation on the generalized seizure activity [83].

ECT primarily affects the prefrontal cortex, hippocampus, and subcortical structures such as the amygdala. The therapy is believed to modulate the limbic system and frontal lobes, which are crucial in mood regulation and cognitive function [84].

The mechanisms of action of ECT include three different phases: (i) the ictal period, characterized by a rise in blood flow, glucose metabolism and oxygen consumption in the cortex; (ii) an interictal period; and (iii) the postictal period, where the therapeutic effect of ECT takes place, reducing the blood flow and the brain glucose metabolism [82].

ECT influences several neurotransmitter systems. It contributes to modifying the metabolism of monoamines, and normalizes the functions of noradrenaline (NA), serotonin (5-HT), dopamine (DA), glutamate, gamma-aminobutyric acid (GABA), and brain-derived neurotrophic factor (BDNF) [85].

The practice of ECT involves administering anesthesia, inducing muscle relaxation and monitoring various vital parameters such as blood pressure, heart rate, oxygen saturation, etc.

Electrodes are placed on the scalp, either unilaterally or bilaterally, to briefly deliver an electrical charge, which induces a seizure that determines the outcome of the treatment [86]. All major international guidelines (APA, CANMAT, RANZCP and WFSBP), recommend ECT as a first, second, third or fourth step in the treatment of several major psychiatric disorders, including BD [87].

ECT treatment typically lasts for about 6 to 12 sessions, administered 2 to 3 times per week during the initial phase. After a significant therapeutic response is achieved, the frequency of treatments can be gradually reduced.

Treatment protocols differ primarily in (i) electrode placement (bitemporal, right unilateral or bifrontal), (ii) electrical dosage (low, moderate or high), and (iii) pulse width (brief or ultra-brief) [82]. Despite the overwhelming effectiveness in mania and depressed states of BD as well as maintenance phases of treatment [83,88], clinicians do not consider ECT earlier in the treatment algorithm.

However, in the about 80-year history of ECT, numerous randomized controlled trials have shown high and rapid rates of improvement or remission for acute mania [88].

Bipolar depression has a statistically higher and faster rate of response than unipolar depression [82]. A better understanding of the factors that predict a higher response to ECT, including influence of baseline medication on ECT outcome, may lead to a better assessment of patients with BD. However, the prognosis may be worse for patients who arrive hostile, agitated, or suspicious [88].

While first-line treatments for mood disorders are ineffective for roughly 60% of young patients, the use of ECT is less common in populations of children and adolescents. Even though ECT is not as commonly used in children and teens, it is still a crucial treatment for children who have serious illnesses like catatonia, affective psychotic disorders, and depression that put them at a high risk of suicide [89]. Additionally, there is growing interest in the use of ECT to treat aggressive and self-harming behavior in individuals with autism spectrum disorder [90]. Children and adolescents tolerate ECT treatment well. Response rates were primarily 70–82% for depression and 87–90% for mania in studies with a sample higher of thirty subjects [91]. Despite its important role in the treatment of psychopathological symptoms, some patients show a transient cognitive impairment [92].

The Mini-Mental State Examination was used in seven trials, and none of them showed evidence of any significant cognitive impairment after the treatment with ECT. The majority of adverse effects were mild and transient. In 1.5% of the cases, ECT was stopped early because of side effects. There were no recorded deaths [91]. Typically, the patient’s response, both in adults and in children, determines the total number of treatment sessions. After two more sessions, ECT is stopped if the patient’s symptoms have improved or not at all [82].

ECT is a safe and effective approach for managing various psychiatric disorders during pregnancy, too, offering therapeutic benefits while minimizing potential adverse effects on both the mother and fetus. This treatment is especially valuable when rapid symptom relief is needed, such as in cases of severe depression with suicidal risk or acute psychosis that impairs the mother’s self-care abilities or poses a danger to herself or others. It is also indicated when symptoms are resistant to pharmacological treatment [93].

Although there are clear benefits to using ECT, there are still some conditions that require further investigation. For example, in clinical practice, psychopathological status may complicate the performance of ECT when patients are unable to provide informed consent [89]; furthermore, the possibility of providing ECT in services dedicated to developmental age is rare and is not yet part of the clinical routine.

A recent literature review on ECT in children and adolescents highlights that, in clinical practice, the use of ECT among minors is infrequent and reserved for rare cases. This may be attributed to the very limited number of psychiatrists (1%) who report having a thorough knowledge of ECT use in adolescents, while the majority of clinicians acknowledge having little to no knowledge or experience with ECT [89].

Another important issue is the stigmatization of ECT by healthcare providers, who still too often do not offer it as treatment, and by patients or family members who are not sufficiently informed about this treatment.

Another important factor is the lack of specific training in ECT, which contributes to international disparities in its use. Training in ECT for child and adolescent psychiatrists is rare, with few universities incorporating the topic into their curricula. However, integrating ECT into the practical training of medical students could significantly improve both knowledge of the technique and attitudes toward the treatment [89].

## 6. Invasive Approaches

### 6.1. Deep Brain Stimulation (DBS)

Deep brain stimulation (DBS) is a neurosurgical procedure involving the implantation of electrodes within specific areas of the brain using stereotactic MRI guidance, followed by the delivery of electrical impulses. Electrodes are connected to a neurostimulator that is implanted near the chest [94].

Contact length, number, and shape are different amongst the DBS electrode configurations. More specifically, a larger contact range increases the variety of possible neural targets, whereas a smaller contact range makes it easier to control stimulation more precisely. The type of DBS system being used determines the different stimulation modes. The current flowing from the battery to the contact, or the other way around, is referred to as “unipolar stimulation”. Current flowing between electrode contacts, at least one of which acts as an anode and the other as a cathode, is indicated as “bipolar stimulation” [95]. The term “interleaving stimulation” describes the switching between various stimulation levels [94].

Originally developed for the treatment of movement disorders such as Parkinson’s disease, DBS has attracted interest as a potential therapeutic option for psychiatric disorders because it may be considered a viable alternative to ablative neurosurgical procedures in certain debilitating mental disorders where this is the case or in cases that do not respond to treatment, such as several forms of obsessive-compulsive disorder (OCD). In addition, it has also been studied in treatment-resistant depression in major depressive disorder (MDD), which has gained increasing importance as a potential add-on strategy [95,96], as well as in BD [97].

DBS involves the implantation of electrodes in specific brain regions, such as the subthalamic nucleus (STN), globus pallidus internus (GPi), and ventral capsule/ventral striatum (VC/VS) [98]. These areas are part of the basal ganglia-thalamocortical circuits involved in motor control, mood, and reward processing. DBS alters the activity of various neurotransmitter systems depending on the target area. For example, stimulation of the STN can modulate dopaminergic pathways, crucial for its effects on Parkinson’s disease. Similarly, DBS in the VC/VS region can influence serotonin and dopamine levels, contributing to its efficacy in treating refractory depression and obsessive-compulsive disorder (OCD) [94,95,99].

The mechanism of action of DBS in BD is complex and not fully understood. However, it is hypothesized to involve the modulation of neural activity, including blood flow, oscillatory synchrony, and synaptic weighting [100], within targeted brain regions, in one or both hemispheres according to the laterality of the symptoms (in movement disorders), such as the subcallosal cingulate cortex (SCC), the ventral striatum, the nucleus accumbens (NAcc), the supero-lateral branch of the medial forebrain bundle (sIMFB), and subcallosal cingulate white matter (Cg25WM) [98,101]. By modulating neural activity in these regions, DBS may normalize dysfunctional circuits involved in mood regulation and emotional processing, too. The primary aim of DBS in BD is to modulate aberrant neural circuits implicated in mood dysregulation, with the goal of reducing symptom severity and enhancing mood stability [101]. An important difference between the DBS application is the open- or closed-loop paradigm. “Open loop” DBS is the current standard in use. The patient receives a single pattern of stimulation to the brain for a few weeks to months after the clinician has examined the medical record and established the stimulation parameters. Indirect behavioral assessment and subjective physician evaluations are the main sources of decision-making in this practice, which heavily relies on trial and error. “Closed loop” DBS identifies a neural biomarker that encapsulates a critical component of disease. After that, the DBS system measures the biomarker directly and makes use of the data to modify the stimulation parameters. Currently manufactured DBS systems can detect local field potentials (LFPs) from electrode contacts at the stimulation site. Prediction algorithms can be used to modify stimulation parameters to obtain the desired neurophysiological signature [100].

In their letter to Editor, Torres et al. [102] firstly reported a 78-year-old woman suffering from bipolar depression who was successfully treated with DBS targeting the subcallosal cingulate region. This approach was chosen due to the poor response observed when treating the patient with various mood stabilizers, antipsychotics, and antidepressants, either alone or in combination.

Moreover, several admissions to the hospital for electroconvulsive therapy (a total of 42 sessions) were required, with a limited clinical response. Therefore, she was considered for DBS. From the first month of stimulation, the patient presented a significant and progressive clinical response supported by a significant reduction in her scores on the Hamilton Depression Rating Scale, the Beck Depression Inventory, and the Montgomery-Asberg Depression Scale, and an increase in her Global Assessment Functioning scores, which persisted at the last follow-up at nine months. No relapse of manic symptoms occurred, as reflected in her scores on the Young Mania Scale.

One review [97] (7) identified a few studies with a total of 12 patients who were treated for bipolar depression with DBS, generally presenting improvement in depressive symptomatology. The target sites were subcallosal cingulate, ventral capsule/ventral striatum, nucleus accumbens, and the supero-lateral branch of the medial forebrain bundle.

The largest study was a 24-week, prospective observational trial [103](7) in which seven patients with BD II depression and ten with major depressive disorder received add-on treatment with DBS of subcallosal cingulate white matter. During the study, BD patients were carefully assessed for hypomanic symptoms and no hypomanic effects occurred in BD patients and antidepressant effects in BD patients were comparable to those in patients with unipolar depression. All seven BD patients had previously undergone ECT, which did not produce the desired effects.

A single bipolar patient, who experienced only one previous manic episode, was included in another study [104] which investigated the use of DBS of the ventral capsule/ventral striatum for treatment refractory depression. This patient’s clinical course was the most variable. Although the patient had periods of depressive symptom improvement lasting weeks, not previously achieved after aggressive treatment including bilateral ECT and VNS, these were followed by hypomanic episodes. Such episodes ceased rapidly upon discontinuation of stimulation, and it is worth noting that hypomanic symptoms can emerge, in general, as a side effect of DBS even in patients suffering from unipolar depression or movement disorders.

Crowell et al. [105] published an open-label, long-term follow-up to examine participants enrolled in a clinical trial of subcallosal cingulate DBS for treatment-resistant depression. Among these patients, seven were diagnosed with bipolar disorder type II, and one patient, initially in the group of participants with major depression, was subsequently reclassified as bipolar type II following an episode of hypomania presented a few years after inclusion in the study. This episode was associated with a change in psychopharmacological therapy and no change in DBS parameters; so, it did not require interruption of stimulation or its modification but only rescheduling of pharmacotherapy.

Among the eight BD patients, five showed a favorable response pattern, and three exhibited limited antidepressant response over time. Four patients in the BD group withdrew from the study, accounting for four of the five participants who withdrew from the study overall.

Most of the above studies, except for the study by Torres et al. [103], included patients with BD II. Bringing additional knowledge on DBS and BD I, a case series of Graat et al. [106], presented five patients with OCD in comorbidity with BD I (three patients) and BD II (two patients), who received DBS of the ventral anterior limb of the internal capsule, to examine the impact on depressive symptoms and OCD. DBS effectively treated symptoms of OCD in four of five patients and significantly reduced depressive symptoms. A focus must be placed on the fact that four of the five patients presented symptoms of hypomania. In three patients (two with BD I and one with BD II) the symptoms resolved spontaneously, while in the fourth (BD II) the symptoms persisted for a long period and required changes in stimulation. Finally, two of the five patients presented suicide attempts, which could be due to a side effect of DBS known as DBS-induced impulsivity. Further studies are therefore needed to better define the risk of suicidality following DBS in BD [104].

Habenula (Hb) has recently been identified as a new DBS target for treating various psychiatric disorders, including depression [96]. Hb is a small bilateral epithalamic structure located adjacent to the posterior commissure in humans, and multiple cortical and cerebellar regions are functionally connected to Hb, which plays a key role in controlling dopaminergic, serotonergic and noradrenergic systems, thus regulating a wide range of behavior beyond reward processing and depressive symptoms. Moreover, according to an MRI study of habenula volume in BD patients, it was found that in those untreated for at least, two months, habenula volume was reduced compared with healthy controls [107].

Two recent reviews [96,108] have attempted to provide an overview of the current status of habenula DBS in human subjects for the treatment of neuropsychiatric disorders. Treated conditions included refractory depression, BD, OCD, schizophrenia, and major depressive disorder. Regarding BD, Zhang and coworkers [109] described the first case of refractory BD successfully treated with habenula DBS. This was a BD I patient who had been suffering from severe depressive symptoms for 4 years and was no longer responding to therapies, including electroconvulsive therapy. Interestingly, after an initial clinical improvement achieved in the first 3 months of high-frequency stimulation, the patient presented with a depressive relapse that improved at 7 months with discontinuation of stimulation. At 8 months, low-frequency stimulation was started, which led to further improvement in quality of life and energy levels. This suggests how low-frequency DBS habenula may be a useful maintenance therapy in bipolar depression. In addition, the patient reported no manic episodes or alterations in cognitive function. In another study, Zhang et al. [110] investigated the habenula’s transient responses to deep brain stimulation. Eight deep brain stimulation contacts were used to test four patients (two of whom had schizophrenia and the other two had bipolar disorder). One month following deep brain stimulation surgery, the patients underwent transient electrical stimulation examinations. The voltage increased in steps of 1 V, ranging from 0 V to a maximum of 10 V. The pulse width was 60 μs. Two frequencies of stimulation, 60 and 135 Hz, were applied to each patient. Out of the 385 active trials, 221 of them produced effects that were stimulated. Pain, heart-rate fluctuations, and numbness were the most frequent temporary side effects. The incidence of pain, involuntary movements, numbness, and changes in heart rate increased as the stimulation voltage increased. All body parts except the scalp experienced numbness due to contralateral stimulation.

In pediatric populations, DBS is currently used for serious neurological disorders such as dystonia, although there are no validated eligibility criteria for DBS in pediatric mental disorders.

Despite its potential, DBS is associated with several limitations. One limitation is the invasiveness of the procedure, as it requires surgical implantation of electrodes within the brain. Additionally, DBS carries risks such as infection, hemorrhage, and device malfunction. Furthermore, response rates to DBS therapy in BD vary among individuals, and not all patients experience significant symptom improvement [101].

However, DBS also offers several advantages. It is a reversible and adjustable treatment option, allowing for fine-tuning of stimulation parameters to optimize therapeutic efficacy. Moreover, DBS may represent a viable treatment option for BD patients who have not responded to traditional pharmacological or psychotherapeutic interventions [96,99].

Evidence regarding the efficacy of DBS in BD is still limited. While some case reports and small-scale studies have reported significant reductions in depressive and manic symptoms following DBS therapy, larger randomized controlled trials with longer follow-up periods are needed to establish its efficacy conclusively [99].

### 6.2. Vagus Nerve Stimulation (VNS)

Vagus nerve stimulation (VNS), developed as an invasive neuromodulation treatment, is a neuromodulation technique that involves applying electrical impulses to the vagus nerve, a major component of the parasympathetic nervous system. American neurologist James Corning is credited with first developing VNS to treat epilepsy in the 1880s. His theory, which relied on data suggesting increased cerebral blood flow as the cause of seizures, was largely shelved for a few years due to conflicting results but resurfaced in the 20th century. The direct effects of VNS on the central nervous system (CNS) were studied by Bailey and Bremer in the 1930s, while Corning focused on the indirect physiological effects of VNS. The results of these studies suggest that VNS alters electroencephalograms (EEGs). For the rest of the century, VNS studies were conducted in animals, but it was not until the 1990s that they entered in clinical trials. The first report of a VNS device implanted in a human was published in 1988 and the first implantable VNS device for the treatment of refractory epilepsy was approved by the FDA in 1997. The use of VNS for depression, migraines, cluster headaches, and obesity in the abdomen has since been approved by the FDA [111].

The approaches to the targeted treatment of VN must be different depending on the indication. In most cases of VNS, a pulse generator is surgically implanted to stimulate the left cervical VN. To reduce cardiac effects, including bradycardia, the left cervical VN is targeted rather than the right. Most often in the context of heart failure, the right cervical VNS was used.

During the initial experiments, the right cervical VN was connected to a programmable device that was implanted in the chest. By preferentially activating vagal efferent fibers, this device was intended to influence cardiac function. Electrodes placed on the ventral and/or dorsal vagal trunks below the diaphragm can also be used to target the subdiaphragmatic network. This approach has been used to study the treatment of obesity and its effects on food intake.

In contrast to invasive implantable VNS devices, transcutaneous VNS (tVNS) is a non-invasive method. To target the auricular branch of the VN, surface electrodes are typically placed on the outer ear. Only the outer ear is the destination of the auricular vagus nerve, the nerve’s peripheral branch [112].

The FDA approved a non-invasive VNS (tVNS) device that is applied to the neck for migraine and cluster headaches, as well as an implanted cervical VNS device that includes an external remote control, lead wire, and pulse generator for treating epilepsy and depression, including BD [113].

The primary aim of VNS in the context of BD is to modulate neural activity in regions implicated in mood regulation, thereby reducing symptom severity and enhancing mood stability.

One of the main reviews of VNS in BD treatment [113] showed the results of 10 weeks to one year of treatment of VNS at 20–30 Hz for BD I or II patients in the depressive phase who had failed at least two medication trials in the current depressive episode.

An observational registry study with a 5-year prospective, open-label, nonrandomized design was carried out at 61 sites in the United States and comprised 795 patients who had gone through four or more unsuccessful attempts at treating their depression, including electroconvulsive therapy (ECT), or who were going through a major depressive episode [unipolar or bipolar depression, 134 subjects with a diagnosis of BD (87 BD I and 47 BD II)], that lasted at least two years. Individuals with rapid cycling bipolar disorder or a history of psychosis were not enrolled. A reduction of at least fifty percent in the initial Montgomery Åsberg Depression Rating Scale (MADRS) score at any postbaseline visit within the five-year study period was considered the primary efficacy measure, known as response rate. Remission was one type of secondary efficacy measure. A significantly higher 5-year cumulative response rate (67.6% vs. 40.9%) and a significantly higher remission rate (cumulative first-time remitters, 43.3% vs. 25.7%) are among the clinical outcomes that the adjunctive VNS group had over the treatment-as-usual group, according to the registry results. According to a sub-analysis, patients in the adjunctive VNS group had a significantly higher 5-year cumulative response rate (71.3% vs. 56.9%) than patients in the treatment-as-usual group among patients who had previously responded to ECT. Not responding to ECT showed a comparable substantial response difference. Regarding efficacy results for treatment-resistant depression, this registry is the longest and largest naturalistic study to date [114].

As Mutz (2023) [26] also reports in his review, VNS has shown promise as a treatment for maintaining symptomatic remission in patients with BD, even when characterized by rapid cycles.

The mechanism of action of VNS in BD is not fully elucidated, but it is thought to involve modulation of neurotransmitter systems and neural circuits involved in mood regulation [115]. Preclinical studies suggest that VNS may enhance the release of neurotransmitters such as serotonin and norepinephrine while also modulating activity in limbic and prefrontal cortical regions implicated in mood disorders [116].

In addition, the effects of the VNS mechanisms of action can be classified as acute or chronic effects. Using oxygen-15-labeled water PET, acute VNS was shown to alter mean cerebral blood flow in several areas associated with depressive states. The right superior and medial frontal cortex, bilateral orbitofrontal cortex, bilateral anterior cingulate cortex, and bilateral temporal cortex all showed an increase in blood flow, while the bilateral temporal cortex and right parietal area showed a decrease. In addition, using BOLD fMRI to measure blood oxygen levels, the long-term effects of VNS were examined [117] 

VNS induced activation in the right anterior insular cortex and the right medial prefrontal cortex in the acute state. But after about 30 weeks, this activation gave way to deactivation, which was accompanied by a decrease in depressive symptoms [111].

Despite its potential, VNS has several limitations. One limitation is the invasiveness of the procedure (VNS), as it requires surgical implantation of a device to stimulate the vagus nerve, although the non-invasive procedure can be a good alternative [112]. Additionally, response rates to VNS therapy in BD vary among individuals, and not all patients experience significant symptom improvement. Moreover, the precise therapeutic mechanisms of VNS in BD remain incompletely understood [114].

However, VNS also offers several advantages. It is generally well tolerated, with few serious adverse effects reported in clinical trials. Furthermore, VNS may represent a viable treatment option for BD patients who have not responded to traditional pharmacological or psychotherapeutic interventions. In fact, patients who have not responded to at least four adequate trials of depression treatment, including ECT, are recommended by the APA to consider VNS [114].

Evidence regarding the efficacy of VNS in BD is still emerging. While some studies have reported significant reductions in depressive and manic symptoms following VNS therapy, others have yielded more mixed results. In the pediatric population, VNS is used to treat neurological conditions, including drug-resistant epilepsy. Further research, including larger randomized controlled trials with longer follow-up periods, is needed to establish the efficacy of VNS as a treatment for BD conclusively.

## 7. How to Direct Patients to a Neuromodulation Treatment

It is crucial to thoroughly evaluate the patient’s medical history, present symptoms, and prior therapies before beginning any neuromodulation therapy. This evaluation should encompass not only the primary neurological or psychiatric condition but also any comorbidities that may influence treatment outcomes or pose contraindications [32] (see Table 3).

Given the complex nature of neuromodulation therapies, a multidisciplinary approach involving neurologists, psychiatrists, psychologists, nurses, pain specialists, and other relevant healthcare professionals is essential. Collaborative discussions allow for comprehensive treatment planning, consideration of alternative options, and mitigation of potential risks [118].

Not all patients may be suitable candidates for neuromodulation treatments. Establishing clear selection criteria based on clinical guidelines, evidence-based research, and individual patient characteristics is imperative. Factors such as symptom severity, treatment responsiveness, and medical history should guide the decision-making process [26,96,99]. The Food and Drug Administration (FDA) has approved the use of ECT devices as Class II (reclassifying them as moderate risk), restricting their application to the treatment of catatonia or severe major depressive episodes associated with major depressive disorder or BD in patients aged 13 and older who are treatment-resistant or require a rapid response due to the severity of their psychiatric or medical condition. The FDA has decided to reclassify the devices used for ECT from Class III (higher risk) to Class II (moderate risk), as they are intended for the treatment of individuals with major depressive episodes associated with MDD or BD who are “treatment-resistant” and “require a rapid response.” The FDA further asserts that the benefits of ECT outweigh the risks.

Another important aspect concerns research in genomics. Recently, a consortium—the Genomics of Electroconvulsive Therapy International Consortium (GenECT-ic)—was established to collect clinical and genetic data with the aim of identifying biomarkers predictive of response to ECT treatment [119]. Regarding symptomatology, certain factors increase the risk of suicide attempts, thereby necessitating timely therapeutic intervention: having a diagnosis of type I, a predominantly depressive polarity, or an index episode with depressive or mixed features [1]. Furthermore, recent findings suggest the need to identify the “biotype” of individuals undergoing neuromodulation to outline a symptomatic profile and predict responsiveness to TMS [30].

Considering that ECT has been practiced for many years, ECT protocols are quite well established. In recent years, specific protocols for its use during pregnancy have also been proposed [93].

The use of TMS requires consideration of a potential adverse effect, namely, seizures. With repetitive TMS (rTMS) protocols, this risk is significantly lower, even among patient populations taking medications that act on the central nervous system; however, it remains an important factor to consider [120].

When multiple factors that could lower the seizure threshold are present, careful consideration should be given to both the clinical indication for TMS and the specific protocol used.

Neurologic conditions involving structural brain damage—such as stroke, multiple sclerosis, traumatic brain injury, Alzheimer’s and other neurodegenerative diseases, and brain infections, tumors or epilepsy—are linked to a heightened risk of seizures. In patients with depression, this risk is particularly elevated in those with dementia or a recent stroke. Additional predictors of seizure risk in depression include low body weight, smoking, alcohol or drug abuse, and the concurrent use of cephalosporins and antiarrhythmics like propranolol [120].

In all these cases, if several temporary risk factors are identified, postponing the TMS session may be an option to consider [120].

Prior to referring a patient for neuromodulation treatment, obtaining informed consent is essential. Patients should be provided with detailed information regarding the proposed therapy, including its mechanism of action, potential benefits, associated risks, and alternative treatment options. Additionally, educating patients about the expected outcomes, realistic expectations, and post-treatment care facilitates active participation in decision-making [24].

Neuromodulation treatments often require specialized expertise and infrastructure for proper administration and management. Referring patients to accredited centers with experienced healthcare providers familiar with the specific modality being considered ensures optimal patient outcomes and safety.

Following initiation of neuromodulation treatment, ongoing monitoring and periodic follow-up are essential components of comprehensive care. Healthcare providers should collaborate closely with patients to assess treatment response, address any emerging concerns or side effects, and make necessary adjustments to the therapeutic regimen [61,118].

## 8. From Research to Practice in the Real-World

Translating neuromodulation techniques for BD from research to clinical practice involves several key steps and considerations. First and foremost, robust empirical evidence from preclinical and clinical studies is essential to establish the safety, efficacy, and mechanisms of action of these techniques in BD. This evidence serves as the foundation for informing clinical guidelines and treatment protocols.

Once the efficacy of neuromodulation techniques for BD has been demonstrated in research settings, the next step is to integrate these techniques into routine clinical practice [121]. This process requires training healthcare providers in the use of neuromodulation technologies, ensuring that they have the necessary expertise to administer these treatments safely and effectively [118].

Additionally, clinicians need to be educated about the appropriate patient selection criteria, the assessment, treatment protocols, and monitoring procedures associated with neuromodulation therapies. Indeed, as mentioned above, it is necessary to adopt specific procedures to monitor the use of substances of abuse, alcohol or psychostimulant medications that may lower the seizure threshold (for example, before proceeding with TMS application), to monitor blood pressure, heart rate, oxygen saturation, etc. (for example, before proceeding with ECT), and to clinically monitor the onset of neurological symptoms related to neurostimulation by TMS, TUS, and others (such as headaches, exacerbation of psychiatric symptoms as hypomania, insomnia, etc.) [42,62]. Furthermore, in invasive techniques, it is essential to monitor the proper functioning of devices implanted in the brain, i.e., for DBS [101](, and the onset of possible cough, dysphonia, and surgical site infection for NVS [111].

Collaboration between researchers, clinicians, and regulatory agencies is crucial for navigating the regulatory approval process and ensuring that neuromodulation techniques for BD meet rigorous standards of safety and efficacy. Regulatory approval is necessary to ensure that these therapies are accessible to patients and reimbursable by healthcare payers.

Furthermore, efforts to disseminate knowledge about neuromodulation techniques for BD among patients, families, and caregivers are essential for promoting awareness and acceptance of these treatments within the broader community. This includes providing accurate information about the potential benefits, risks, and side effects of neuromodulation therapies, as well as addressing any misconceptions or stigma associated with these interventions.

Finally, ongoing research and innovation are necessary to refine existing neuromodulation techniques, develop new treatment modalities, and further elucidate the underlying mechanisms of action of these therapies in BD.

In order to define tailored neuromodulation’s treatments, there is a need to consider the following essential elements.

The devices used should have the ability to stably record and track the individual activity of the neurons within neural circuits in every single instant, a capability that not all current technologies have. Another element to consider is the ability to modulate neurons within neural circuits in response to changes in monitored signals and the ability to implement closed-loop feedback systems that monitor and modulate individual neurons within neural circuits. Finally, monitoring and modulation of specific neuronal subtypes is also essential [121].

Longitudinal studies are needed to assess the long-term safety and effectiveness of neuromodulation interventions, as well as to identify factors that predict treatment response and optimize treatment outcomes.

Another important point to address is the role of the stigma. In fact, reducing the stigma surrounding neuromodulation techniques such as ECT is of paramount importance for their successful translation from research to clinical practice. Stigma can significantly impede patient access to these potentially life-saving treatments and can also influence healthcare professionals’ attitudes and willingness to recommend them [87].

Education and training programs should be implemented to dispel myths and misconceptions about ECT and other neuromodulation techniques. Providing evidence-based information about the safety, efficacy and appropriate use of these treatments can help healthcare providers feel more confident in recommending them to eligible patients.

Similarly, reducing the stigma among the general population is essential for promoting acceptance and accessibility of neuromodulation therapies. Public awareness campaigns, media initiatives, and community outreach efforts can help challenge negative stereotypes and foster a more accurate understanding of these treatments. Personal testimonials from individuals who have benefited from neuromodulation therapies can be particularly impactful in humanizing these interventions and reducing fear and apprehension [122].

Furthermore, advocacy efforts aimed at policy-makers and healthcare administrators can help ensure that adequate funding and resources are allocated for the provision of neuromodulation treatments. This includes advocating for insurance coverage and reimbursement policies that enable equitable access to these therapies for all individuals who could benefit from them [118].

By combating stigma and promoting informed understanding of neuromodulation techniques like ECT or VNS, we can create a more supportive and inclusive environment for individuals with BD and other psychiatric conditions. This, in turn, can facilitate their timely access to evidence-based treatments and ultimately improve their quality of life and treatment outcomes.

## 9. Training for Clinicians

First and foremost, robust empirical evidence from preclinical and clinical studies is essential to establish the safety, efficacy, and mechanisms of action of these techniques in BD. This evidence serves as the foundation for informing clinical guidelines and treatment protocols.

Once the efficacy of neuromodulation techniques for BD has been demonstrated in research settings, the next step is to integrate these techniques into routine clinical practice. This process requires comprehensive training of mental health practitioners in the use of neuromodulation technologies, ensuring they have the necessary expertise to administer these treatments safely and effectively. Providing continuing education opportunities can help practitioners stay updated on the latest advancements in neuromodulation therapies [118].

Additionally, it is crucial to empower patients and the general population with knowledge about neuromodulation techniques for BD. This includes raising awareness about these treatment options, debunking myths and misconceptions, and providing accurate information about their potential benefits, risks and side effects. By empowering patients to make informed decisions about their treatment options, we can promote shared decision-making and improve treatment adherence and outcomes.

Moreover, efforts to disseminate knowledge about neuromodulation techniques among patients, families, and caregivers are essential for promoting acceptance and reducing stigma associated with these interventions. Providing support groups, educational materials, and peer counseling can help individuals navigate their treatment journey and access appropriate care [122].

Collaboration between researchers, clinicians, patient advocacy groups, and regulatory agencies is crucial for navigating the regulatory approval process and ensuring that neuromodulation techniques for BD meet rigorous standards of safety and efficacy [24]. Regulatory approval is necessary to ensure that these therapies are accessible to patients and reimbursable by healthcare payers.

Finally, ongoing research and innovation are necessary to refine existing neuromodulation techniques, develop new treatment modalities, and further elucidate the underlying mechanisms of action of these therapies in BD. Longitudinal studies are needed to assess the long-term safety and effectiveness of neuromodulation interventions, as well as to identify factors that predict treatment response and optimize treatment outcomes.

## 10. Limitations

Despite their potential, neuromodulation techniques have several limitations. Invasive techniques carry risks associated with surgical implantation, such as infections and device malfunctions. Non-invasive techniques may have limited depth of penetration and may be less effective in modulating deeper brain structures. Additionally, individual response to neuromodulation therapy can vary, and not all patients experience significant symptom improvement.

Although numerous studies on neuromodulation techniques are present in the literature, some lack clear and consistent guidelines for the treatment of BD and many are dependent on the type of device used.

Another significant limitation concerns the translation of knowledge gained from laboratory research on neuromodulation techniques into clinical practice.

As a recent Editorial published on *Nature Biotechnology* [123] reported, there are several obstacles limiting the translation of neuromodulation techniques into clinical practice. First, there is a lack of critical knowledge regarding the mechanisms of action, optimal dosing, response relationships, and how individual differences in neural structure and function may influence the performance of different devices. Another important aspect is the need for public investment in neurotechnology acquisition, which, despite recent cost reductions, still requires dedicated funding for device purchase, clinician training, maintenance, to mention a few. The devices used in clinical practice are evolving, moving from simple, preset stimulation patterns to closed-loop models that monitor the neural environment and respond with tailored, patient-specific stimulation. This shift, on the other hand, requires robust training for clinicians and specialized teams who can develop specific expertise.

This is even more challenging in invasive treatments, where individual differences complicate both the application and monitoring of the device within the brain.

The literature produced to date reveals several critical issues, including challenges with replicability in some studies, the use of sham controls and the frequent occurrence of small sample sizes, which often hinder efforts to correlate stimulus dose with therapeutic response. These factors create obstacles that hinder meaningful progress in this field. This is particularly challenging given the relatively small number of specialized clinical groups dedicated to the development of neuromodulatory devices.

## 11. Conclusions

To our knowledge, this is the first narrative review to integrate the latest neuromodulation strategies with real-world treatment approaches for BD.

Neuromodulation strategies engage complex neural networks and diverse neurotransmitter systems. These therapies offer significant therapeutic benefits for various psychiatric and neurological disorders, including BD, by modulating brain activity in targeted regions.

There are currently no standard neuromodulation treatment methods for the child population, where neuromodulation is often limited to rehabilitation treatment. Therefore, neuromodulation techniques can potentially create new opportunities for both the treatment of psychopathological conditions and neuromodulation to promote brain plasticity [22].

Understanding the specific neuroanatomical areas, circuits, and neurotransmitters involved in these treatments is essential for optimizing their efficacy and expanding their clinical applications, above all, in lifelong BD [30,61]. 

Effective referral of patients to neuromodulation treatments necessitates a systematic approach encompassing comprehensive assessment, multidisciplinary collaboration, adherence to selection criteria, informed consent, referral to specialized centers, and continued monitoring [118,124]. By following these best practices, healthcare providers can optimize patient outcomes and enhance the efficacy of neuromodulation therapies in BD, too [24,125].

However, more investigation is required to ascertain the clinical usefulness of neuromodulation interventions in adult and childhood BD. Future clinical trials should aim to determine which specific group(s) of BD patients may benefit from specific neuromodulation treatments [30].

In summary, translating neuromodulation techniques for BD from research to clinical practice requires a multidisciplinary approach, encompassing scientific inquiry, clinical expertise, regulatory oversight, patient education, and ongoing innovation [22]. By leveraging advances in neuromodulation technology and evidence-based practice, we can improve the quality of care and outcomes for individuals living with BD, ultimately reducing the burden of untreated illness and ineffective treatments.

## Figures and Tables

**Table 1:** Pharmacotherapy table for the management of the different phases of BD. (*LAI: long acting injection*).

*Phases*	Acute Manic/Mixed Episodes	Depressive Phase	Euthymic/ Maintenance
**Childhood**	Lithium + Atypical Antipsychotics (*risperidone*, *olanzapine*, *quetiapine*, *aripiprazole*, *ziprasidone*, *and asenapine*)	Olanzapine and Fluoxetine or Lurasidone (*as monotherapy*)	Aripiprazole Lamotrigine (*in adolescents only*) Lithium
**Adulthood**	Haloperidol Atypical Antipsychotics (*risperidone*, *olanzapine*, *quetiapine*, *aripiprazole*, *ziprasidone*, *asenapine*, *paliperidone and cariprazine*) + BDZ Lithium Anticonvulsant (*carbamazepine*, *valproate*)	Atypical Antipsychotics (*quetiapine*, *olanzapine*, *cariprazine*, *lumateperone*, *and* *lurasidone*) Antidepressant or SSRI Anticonvulsant (*valproate*, *lamotrigine*) Lithium	Lithium Antidepressant or SSRI Anticonvulsant (*valproate*, *lamotrigine*) Atypical Antipsychotics (*risperidone LAI*, *olanzapine*, *quetiapine*, *aripiprazole*, *and asenapine*)

**Table 2:** Psychosocial treatment scheme for the management of the different phases of BD [CBT: Cognitive and Behavioral Therapy; FFT: Family Focus Therapy; IPSRT: Interpersonal and Social Rhythm Therapy].

*Effective for Management During*	Acute Manic/Mixed Episodes	Depressive Phase	Euthymic
**Childhood**	CBT (*medication-adherence-oriented*)	CBT (*on the management of depression symptoms*)	Psychoeducation (*enhanced relapse prevention*) individual/group FFT
**Adulthood**	CBT (*medication-adherence-oriented*)	CBT (*on the management of depression symptoms*)	Psychoeducation (*enhanced relapse prevention*) individual/group FFT CBT (*on the recognition of early signs of crisis*) IPSRT (*on the life rhythms*)

**Table 3:** Neuromodulation strategies in BD: pros and cons.

**Neuromodulation Strategies in BD: Non-Invasive Approaches**		
	**Pros**	**Cons**
TMS	It is a safe technique, well tolerated and with a low rate of drop-out With rTMS protocols the risk of seizures is significantly low	The seizures as a potential adverse effect In pediatric population the existing research is very limited
TES	It is a well-tolerated technique with few serious adverse effects It can be administered as an adjunctive therapy, potentially enhancing treatment outcomes	Variability in treatment response observed among individuals The optimal parameters have yet to be established The long-term effects remain unclear
TUS	It does not require surgical implantation or anesthesia It is a well-tolerated technique and suitable for repeated sessions It has a high spatial resolution	The lack of a precise targeting of brain regions The optimal parameters have yet to be fully elucidated The long-term safety profile remains to be established
PBM	It is a well-tolerated technique with minimal side effects It is suitable for a long-term use It can be administered and integrated into existing treatment regimens	The lack of standardized protocols regarding treatment parameters The evidence supporting the efficacy is still preliminary with relatively few well-designed clinical studies available
NF	It is a potentially attractive option for individuals seeking non-pharmacological interventions The training protocols can be tailored to target specific symptoms of BD, allowing for individualized treatment approaches	The effects may not generalize well beyond the training setting, limiting its long-term efficacy The training requires multiple sessions over an extended period, making it resource-intensive and potentially inaccessible to some individuals
ECT	It is a safe technique It is an effective approach for managing various psychiatric disorders during pregnancy, offering therapeutic benefits while minimizing potential adverse effects on both the mother and fetus, such as in cases of suicidal risk It is indicated when symptoms are resistant to pharmacological treatment	The lack of specific training which contributes to international disparities in its use The training for child and adolescent psychiatrists is rare
**Neuromodulation strategies in BD: Non-Invasive Approaches**		
	**Pros**	**Cons**
DBS	It is a reversible and adjustable treatment option It may represent a treatment option for patients who have not responded to traditional interventions	The surgical implantation of electrodes within the brain The risks involve infection, hemorrhage and device malfunction The response rates vary among individuals
VNS	It is well tolerated, with few serious adverse effects It may represent a treatment option for patients who have not responded to traditional interventions	The surgical implantation of a device to stimulate the vagus nerve The precise therapeutic mechanism remains incompletely understood The response rates vary among individuals

## Data Availability

No new data were created.
